# Effects of gabapentin on muscle spasticity and both induced as well as spontaneous autonomic dysreflexia after complete spinal cord injury

**DOI:** 10.3389/fphys.2012.00329

**Published:** 2012-08-15

**Authors:** Alexander G. Rabchevsky, Samir P. Patel, Travis S. Lyttle, Khalid C. Eldahan, Christopher R. O'Dell, Yi Zhang, Phillip G. Popovich, Patrick H. Kitzman, Kevin D. Donohue

**Affiliations:** ^1^Spinal Cord and Brain Injury Research Center, University of KentuckyLexington, KY, USA; ^2^Department of Physiology, University of KentuckyLexington, KY, USA; ^3^Department of Neuroscience, Ohio State UniversityColumbus, OH, USA; ^4^Center for Brain and Spinal Cord Repair, Ohio State UniversityColumbus, OH, USA; ^5^Rehabilitation Sciences, University of KentuckyLexington, KY, USA; ^6^Electric and Computer Engineering, University of KentuckyLexington, KY, USA

**Keywords:** neuropathic pain, colorectal distension, power spectral density, telemetry, blood pressure, heart rate

## Abstract

We recently reported that the neuropathic pain medication, gabapentin (GBP; Neurontin), significantly attenuated both noxious colorectal distension (CRD)-induced autonomic dysreflexia (AD) and tail pinch-induced spasticity compared to saline-treated cohorts 2–3 weeks after complete high thoracic (T4) spinal cord injury (SCI). Here we employed long-term blood pressure telemetry to test, firstly, the efficacy of daily versus acute GBP treatment in modulating AD and tail spasticity in response to noxious stimuli at 2 and 3 weeks post-injury. Secondly, we determined whether daily GBP alters baseline cardiovascular parameters, as well as spontaneous AD events detected using a novel algorithm based on blood pressure telemetry data. At both 14 and 21 days after SCI, irrespective of daily treatment, acute GBP given 1 h prior to stimulus significantly attenuated CRD-induced AD and pinch-evoked tail spasticity; conversely, acute saline had no such effects. Moreover, daily GBP did not alter 24 h mean arterial pressure (MAP) or heart rate (HR) values compared to saline treatment, nor did it reduce the incidence of spontaneous AD events compared to saline over the three week assessment period. Power spectral density (PSD) analysis of the MAP signals demonstrated relative power losses in mid frequency ranges (0.2–0.8 Hz) for all injured animals relative to low frequency MAP power (0.02–0.08 Hz). However, there was no significant difference between groups over time post-injury; hence, GBP had no effect on the persistent loss of MAP fluctuations in the mid frequency range after injury. In summary, the mechanism(s) by which acute GBP treatment mitigate aberrant somatosensory and cardiophysiological responses to noxious stimuli after SCI remain unclear. Nevertheless, with further refinements in defining the dynamics associated with AD events, such as eliminating requisite concomitant bradycardia, the objective repeatability of automatic detection of hypertensive crises provides a potentially useful tool for assessing autonomic function pre- and post-SCI, in conjunction with experimental pharmacotherapeutics for neuropathic pain, such as GBP.

## Introduction

Spinal cord injury (SCI) is a serious health care problem in the United States striking, on average, 12,000 individuals each year. Approximately 270,000 Americans are living with the typically devastating neurological deficits and debilitating somatic and autonomic reflexes in chronic SCI (see https://www.nscisc.uab.edu). In particular, complete as well as incomplete SCI above high-thoracic levels can lead to a potentially life-threatening hypertensive condition termed autonomic dysreflexia (AD) that is often triggered by noxious somatic or visceral stimuli below the injury level (Karlsson, 1999). Due to the disruption of descending modulating pathways from the brainstem, this syndrome is characterized by episodic, sympathetically-driven reflexive hypertension which is usually accompanied by intact baroreflex-mediated bradycardia (Rabchevsky, 2006). Episodes of AD often cause debilitating symptoms including pounding headache, acute anxiety, shivering, flushing and sweating (Kewalramani, 1980). One of the most common triggers of AD is the distension of pelvic viscera (bladder and bowel) (Snow et al., 1978; Harati, 1997; Krassioukov et al., 2003).

The development of animal models of noxious colorectal distension (CRD)-induced AD (Krassioukov and Weaver, 1995; Rivas et al., 1995) to mimic clinical manifestations of fecal impaction have enabled the detailed analysis of temporal dynamics of CRD-induced hypertension (Maiorov et al., 1997b, 1998). Accordingly, the development and severity of AD has been correlated with extent of aberrant sprouting of nociceptive C-fibers into the spinal cord below the injury (Krenz et al., 1999; Marsh et al., 2002; Cameron et al., 2006), and glutamatergic neurotransmission has been shown to contribute to spinal viscerosympathetic initiation of episodic hypertension (Maiorov et al., 1997a).

There is currently no single clinical intervention which effectively attenuates the manifestation of both muscle spasticity and AD stemming from SCI (Rabchevsky and Kitzman, 2011). Notably, however, one compound that has been shown to interfere with glutamatergic transmission and is safe for clinical use is gabapentin (GBP, Neurontin®; Pfizer, New York, NY, USA), which is approved for the treatment of epilepsy and is widely used off-label for the treatment of neuropathic pain (Kitzman et al., 2007). Accordingly, we have been testing the hypothesis that GBP can alleviate both spasticity and AD by impeding neurotransmission of noxious stimuli into the spinal cord, thus eliminating a critical physiological link between these aberrant reflexes. To this end, we recently reported that acute GBP administration significantly attenuates both AD and tail spasticity induced by noxious stimuli compared to saline-treated cohorts at 2–3 weeks post-injury (Rabchevsky et al., 2011).

In order to further characterize the effects of GBP and determine whether it can be administered as a prophylactic, here we report the results of long-term telemetry experiments designed to assess (1) whether daily GBP versus saline alters baseline cardiovascular parameters, (2) the efficacy of chronic, daily GBP treatment versus acute administration in modulating experimentally-induced AD and tail spasticity over 3 weeks post-injury, and (3) whether daily GBP modulates spontaneous AD events detected using a novel algorithm we have developed based on blood pressure telemetry data.

## Materials and methods

### Surgical methods; implantation of blood pressure telemetry devices in descending aorta

As described in detail (Rabchevsky et al., 2011), seven days prior to T4 transection SCI, naïve anesthetized (ketamine, 80 mg/kg, i.p.; xylazine 7 mg/kg, i.p.) rats (*n* = 12) were implanted with telemetric pressure transmitters (model TA11PA-C40, Data Sciences International, Inc., St. Paul, MN) into the descending aorta after its brief occlusion and securing the probe to the abdominal wall with silk sutures. The skin was closed with surgical staples after rinsing abdominal cavity with saline. The animals were then treated post-operatively, as described below, and blood pressure was monitored 24/7 to ensure patency of the probes and to obtain pre-injury baseline data.

### Surgical methods; spinal cord injury

All surgical procedures were performed under aseptic conditions using sterilized instruments, following the University of Kentucky IACUC and the NIH guidelines. One week following telemetry implantation, the T4 spinal segment of anesthetized (ketamine, 80 mg/kg, i.p.; xylazine 7 mg/kg, i.p.) female Wistar rats (~225 g) was exposed by T3 laminectomy (*n* = 12) and the spinal cord was completely transected with a scalpel blade before hemostasis was achieved with gelfoam placed into the resection site, as previously detailed (Cameron et al., 2006; Rabchevsky et al., 2011). Wounds were then irrigated with sterile saline, the muscles sutured using 3-0 vicryl and skin openings stapled with wound clips. Injured rats were housed one per cage with food and water *ad libitum*, placed on a heating pad during recovery, and injected with 10 ml Lactated Ringer's solution s.c. for fluid replacement. Upon regaining consciousness, post-surgery pain was alleviated by administering buprenorphine (0.02–0.05 mg/kg, s.c., Reckitt Benckiser, Hull, UK) twice a day for three days. The injured animals required manual bladder evacuation twice a day for 2–3 weeks post-injury until spontaneous bladder voiding returned with no signs of urinary tract infection. They also received twice daily injections (s.c.) of antibiotics (33.3 mg/kg Cefazolin, s.c., SoloPak Laboratories, New Gove, IL) and Ringer's for 5 days.

### Telemetric monitoring of blood pressure before and after SCI

Following transection SCI, the Dataquest A.R.T. system (Data Sciences International, Inc., St. Paul, MN) was used for 24/7 telemetric monitoring of pulsatile arterial blood pressure (PAP), as well as on-demand monitoring of blood pressure prior to, during, and after noxious CRD. CRD was performed on four separate days following SCI. Specifically, on days 14, 15, 21, and 22, injured animals in both chronic treatment groups were injected with either acute GBP or saline 1 h prior to cardiophysiology in response to CRD (see Table 1). For each gently restrained conscious rat, a period of 15 min was allowed to pass to allow them to become quiet after carefully inserting a latex balloon-tipped catheter (Swan-Ganz Paceport catheter; Baxter Healthcare Corporation, CA) 2 cm inside the rectum and securing it to the tail with tape. Prior to each initiation of spinal viscero-sympathetic reflexes, 30 s of baseline arterial pressure was recorded. Measurements continued during the gradual (15 s) inflation of the 10 mm long balloon inflation (CRD) with 2 ml of air for a period of 60 s, followed by another 30 s following balloon deflation. Such CRD expands the colon as would several large fecal boluses. During a recording period, animals within individual cages were placed upon receiver plates and PAP readings were transmitted to a receiver (PhysioTel Receiver, RPC-1 from Data Sciences International) as a radio frequency signal integrated by a data exchange matrix. The two traces for each animal trial were saved and stored in a selected file for subsequent analyses. Thus, two separate trials were conducted for each animal separated by ~30 min on each of the four testing days. The average MAP and HR values over the entire dynamic 60 s CRD period were calculated prior to averaging the values over the two trials for each animal; the mean MAP and HR values across each treatment group were then derived from summing individual animal averages.

**Table 1:** **Quantified changes in mean arterial pressure (MAP) and heart rate (HR) measurements from baseline during one minute of noxious colorectal distension (CRD) in injured rats treated chronically (daily morning) with either Saline or GBP**.

**Acute administration (Time points)**		**Chronic treatment groups**			
		**MAP (mmHg)**		**HR (beats per min)**	
		**Saline**	**GBP**	**Saline**	**GBP**
Saline	14 DPI	19.7 ± 2.5	–	−43.8 ± 12.2	–
GBP		–	9.0 ± 3.6^*^	–	4.1 ± 10.9^*^
Saline	15 DPI	–	26.3 ± 3.8	–	−13.7 ± 10.1
GBP		5.9 ± 1.1^***^	–	−16.0 ± 4.6	–
Saline	21 DPI	38.0 ± 3.4	–	−36.1 ± 19.5	–
GBP		–	14.7 ± 4.7^**^	–	−16.3 ± 15.5
Saline	22 DPI	–	34.3 ± 3.5	–	−50.9 ± 22.0
GBP		19.4 ± 3.1^*^	–	−6.8 ± 7.2	–

Both chronic treatment groups were subjected to CRD after either Acute GBP or Acute Saline injections prior to assessments on alternating days (days 14–15 and 21–22). Acute GBP treatment significantly reduced CRD-induced MAP increases by approximately two-fold compared to Acute Saline treatment, irrespective of Chronic (daily morning) Treatment Groups. While bradycardia appeared to be reduced at all time points examined, the variability precluded significant differences. Values represent group means ± SEM.

* p ≤ 0.05,

** p ≤ 0.005 and

*** p ≤ 0.001 between Acute GBP versus Acute Saline groups at all Administration Time Points.

### Behavioral assessment for spasticity in the tail muscles

Between the two trials for each of the four separate days that CRD was performed (~30 min), spasticity in the tail was assessed behaviorally using an established scale (Kitzman, 2006; Kitzman et al., 2007). Specifically, the response of the tail muscles to a quick stretch, light stroking (non-noxious stimulus) and a light pinch (noxious stimulus) applied approximately 10 cm from the tip of the tail were assessed. Tail manipulations were performed with the animals lightly restrained and the tail was free to move over its full length. In this study, the animals displayed either a stage-2, stage-3, or stage-4 spasticity prior to initiating pharmacological intervention (see Table 2). For the purpose of statistical analysis, responses to quick stretch and pinch were graded using a five point scale in which 0 = minimal (≤45° flexion) response to the stimulus, 1 = 45–90° flexion, 2 = >90–180° flexion, 3 = >180–225° flexion, 4 = >225–360° flexion, and 5 = significant coiling of the tail and/or activation of flexors, extensors, and abductors (writhing) lasting >2 s and the presence of clonus. The response to light touch was scored using a three point grading scale in which 0 = no response, 1 = minimal flexion of the tail away from the stimulus, and 2 = pronounced flexing of the tail away from the stimulus.

**Table 2:** **Behavioral responses of the tail musculature 2–3 weeks following T4 spinal cord transection (same rats as in Table 1) in response to Touch (Left), Stretch (Middle) and Pinch (Right) 1 h following the administration of Saline or GBP**.

**Acute administration (Time points)**		**Chronic treatment groups**					
		**Saline**	**GBP**	**Saline**	**GBP**	**Saline**	**GBP**
		**Tail touch**		**Tail stretch**		**Tail pinch**	
Saline	14 DPI	2.0 ± 0.2	–	5.0 ± 0.2	–	5.0 ± 0.0	–
GBP			–	0.0 ± 0.2^*^	–	0.0 ± 0.4^*^	–	1.0 ± 0.4^*^
Saline	15 DPI	–	2.0 ± 0.0	–	5.0 ± 0.4	–	5.0 ± 0.6
GBP		0.0 ± 0.2^*^	–	0.0 ± 0.3^*^	–	0.0 ± 0.2^*^	–
Saline	21 DPI	2.0 ± 0.2	–	5.0 ± 0.0	–	5.0 ± 0.0	–
GBP		–	0.0 ± 0.2^*^	–	0.0 ± 0.4^*^	–	0.0 ± 0.4^*^
Saline	22 DPI	–	2.0 ± 0.0	–	5.0 ± 0.0	–	5.0 ± 0.0
GBP		0.5 ± 0.3^**^	–	0.0 ± 0.8^**^	–	0.0 ± 0.8^**^	–

Response to Tail Touch was scored on a 2 point scale in which 0 = no spasticity and 2 = severe spasticity. Responses to Tail Stretch and Tail Pinch were scored on a 5 point scale in which 0 = no spasticity and 5 = severe spasticity. Quantitative analyses confirmed the striking effect of Acute GBP treatment in virtually abolishing all three measures of tail spasticity, irrespective of the Chronic (daily) treatment over days post-injury (**DPI**).

* p < 0.05 Values represent group medians ± SEM for visualization purposes only.

### Drug administration

Every morning at 9:00 am, beginning the day after SCI, animals received a daily i.p. injection of either GBP (50 mg/kg; Neurontin) or saline vehicle. Notably, however, on days 15 and 22 the treatment groups were reversed to assess the effects of acute versus chronic GBP administration on the cardiophysiological responses to CRD and tail manipulations (see Tables 1 and 2). Importantly, the half-life of GBP is 5–9 h in humans, which is unaltered following multiple dosing (Goa and Sorkin, 1993; McLean, 1995). Accordingly, when a person receives GBP 3–4 times per day, the half-life is still reported as being 5–9 h; thus the required multiple dosages each day. If the half-life altered with multiple dosages, then once a patient reached the therapeutic dosage (typically 3–4 dosages per day), over time one might expect to be able to decrease the number of dosages per day since the therapeutic half-life would increase. However, this does not appear to be the case with GBP.

### Detecting spontaneous incidences of autonomic dysreflexia

**Figure 1:**
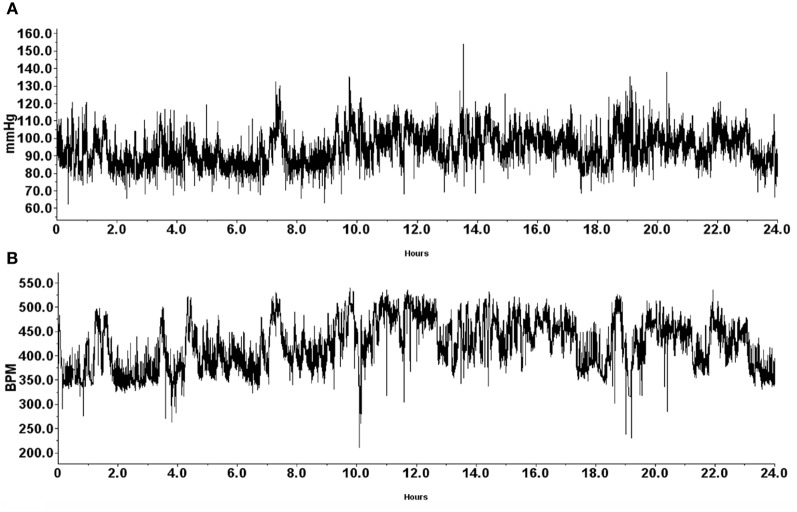
**Representative traces of mean arterial pressure (A; mmHg) and heart rate (B; BPM) recorded from a naïve animal 1 day prior to T4 spinal cord transection.** Data was gathered telemetrically over a 24-h period using an implantable probe that was inserted into the descending aorta. Animals were housed one per cage to allow for individual telemetric monitoring. Receivers placed beneath each animal's cage telemetrically relayed signals into a data exchange matrix, where the signal was integrated into files to be opened with the Dataquest A.R.T. software package for subsequent analyses.

An algorithm was developed to automatically detect spontaneous AD events based on the 24 h blood pressure and heart rate (HR) telemetry data. Concurrent values for mean arterial blood pressure (MAP) and HR were recorded using DataQuest (Data Sciences International, Inc., St. Paul, MN) at 2 s intervals for 4 days pre-injury and 22 days following T4 spinal cord transection. Example waveforms of MAP and HR are shown in Figures 1 and 4. The algorithm processed these waveforms to effectively estimate the number of instances where an abnormally sharp MAP increase was accompanied by HR decrease, as illustrated in Figure 3. This was implemented with a program written in Matlab (The MathWorks, Inc., Natick, MA).

The MAP and HR signals were initially low-pass filtered at a 0.04 Hz cut-off frequency with a 6th order Butterworth filter to smooth out transient spikes (less that 12.5 s duration) and limit their impact on threshold crossings. A baseline comparison for MAP was created by a moving average window of 240 s. This was used for comparing the MAP values at 25 s after the end of the averaging window. Note that in Figure 3, the MAP values were delayed by 25 s relative to the baseline average so comparisons could be more easily visualized. A MAP peak was associated with a detected AD event when simultaneous numerical conditions were met, as illustrated in Figure 3. The following are the conditions with values used in the algorithm for results presented in this report:

1. MAP peak exceeds the baseline by *T*_*p*_ = 10 mm Hg or greater.
2. The difference between the MAP peak value and the MAP minimum value within the previous *T*_*r*_ = 35 seconds must be *T*_*s*_ = 20 mm Hg or greater than this peak (i.e., MAP swing must be sufficiently large and fast).
3. The HR must drop by *T*_*h*_ = 10 bpm or greater within the MAP event interval, defined as staring with the MAP exceeding *T*_*p*_ and ending when it drops below this same value (see example in Figure 3A). The maximum HR associated with the drop must occur within the first 75% of this interval and the minimum HR value must occur after this maximum value and within 5 s beyond the end of the MAP interval (i.e., the HR drop must be sufficiently close to the elevated MAP event).

### Spectral analysis

To examine persistent MAP dynamics between groups, a power spectral density (PSD) analysis was performed. This analysis complements the result for the spontaneous AD events in that it detects differences in persistent dynamics, since it averages over the observation interval. Sparse transient events are averaged out in this case. The PSDs were computed using Welch's method (Proakis and Manolakis, 1996) from data recorded for 4 h following the daily injections. The raw pulsatile blood pressure signal was originally sampled at 512 Hz, and re-sampled to 50 Hz after an anti-aliasing filter was applied. Spectral magnitudes were computed from 128 s segments with linear trends removed, and a 50% overlap was used between consecutive segments.

### Spinal cord tissue processing and histology

After final analyses, all injured rats were overdosed with sodium pentobarbital and transcardially perfused with 4% paraformaldehyde in PBS. Dissected spinal cords from T4-transected rats were stored for long term storage at 4°C in 20% sucrose/PBS containing 0.02% sodium azide.

### Statistics

All data were both collected and analyzed in a blinded manner, as routinely performed in our published pharmacological studies. Notably, all analyses were performed by individuals blinded with respect to treatment. For comparisons of CRD-induced MAP and HR changes, unpaired Student's *t*-tests, with bonferroni correction factor when appropriate, were used between saline- and GBP-treated groups. Specifically, we compared the average MAP and HR values over the entire 60 s CRD period, and averaged the values over the two trials for each animal. To compare AD events over time between groups, a repeated measures analysis of variance (ANOVA) was performed, followed by Fischer's PLSD when appropriate. Each behavioral test (tail responses to stretch, noxious pinch, and non-noxious light touch, as well as presence of clonus) was compared using the Mann-Whitney U test for ordinal data. PSD data were analyzed via power ratios between low and mid frequencies ranges and 95% confidence limits for each group were computed. Statistical significance was set *a priori* at *p* < 0.05 for all analyses.

### Validation of algorithm

The parameters of the AD algorithm were optimized using a data set consisting of 156 h of data labeled by an observer. The level of agreement between human observation and the automatic algorithm was assessed by estimating the probability of agreement on humanly detected AD events (47) from 169 h of telemetry data from 3 SCI rats. The results are described by two metrics, a probability of positive agreement (when the algorithm and observer identified the same time segment as containing an AD event), as well as a negative agreement rate (how often per second the algorithm identifies an AD event when the human observer did not). The test resulted in a positive agreement probability of 0.87 and a negative agreement rate of 6.0 × 10^−5^ per second (approximately five detections per day). In addition, the algorithm was applied to 72 h of pre-injury data (where no AD events are expected) and resulted in two detections per day, which is considered a false-detection rate of 2.3 × 10^−5^ per second. Given the expected level of human error, especially for large data sets, this is considered a good agreement.

## Results

### Effects of daily GBP treatment on resting blood pressure and heart rate

**Figure 2:**
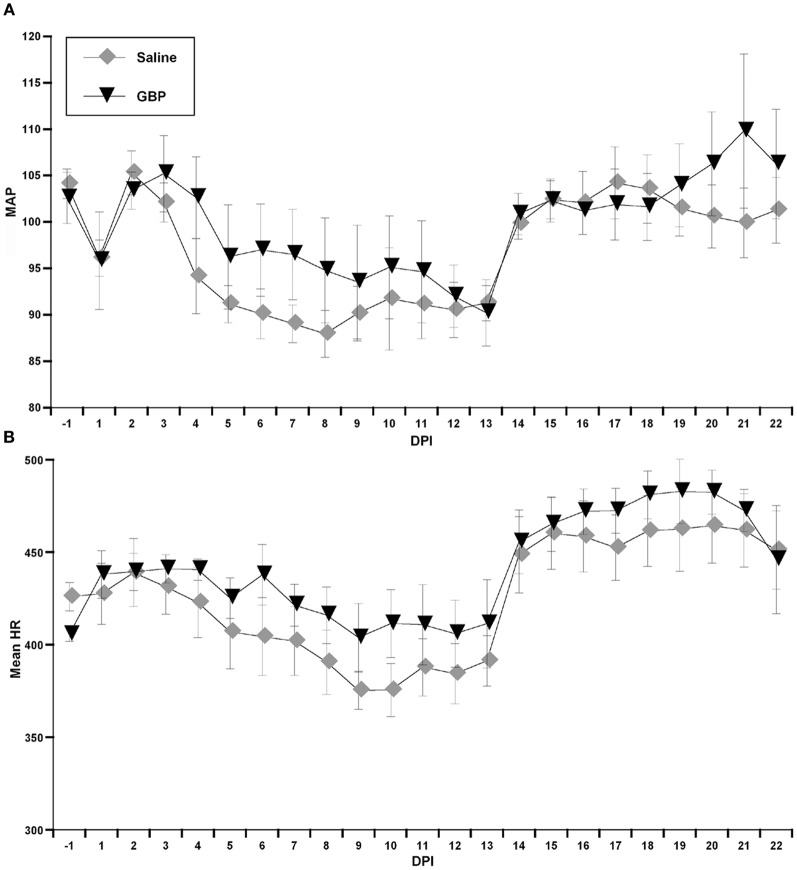
**Graphs representing both mean arterial pressure (A, MAP) and heart rate (B, HR in beats per minute) across days post-injury (DPI) gathered telemetrically over 24 h periods, beginning at 9:00 am following injections of either Saline or gabapentin (GBP).** Due to the overall variability in both outcome measures, there were no significant differences between treatment groups for daily MAP and HR values. The conspicuous sustained elevation in both daily MAP and HR values beginning on days 14–15 post-injury that approached pre-injury values are interesting. *n* = 6 Saline; *n* = 5 GBP (50 mg/kg). Symbols represent group means ± SEM error bars.

During the experiment, one GBP-treated animal died shortly after surgery for uncertain reasons. All animals were housed one per cage and Figure 1 demonstrates blood pressure recordings gathered telemetrically from a rat prior to SCI over a 24 h period. Every morning, beginning the day after SCI, the injured animals received an injection (i.p.) of either GBP or saline vehicle. As depicted in Figure 2, assessments over 24 h periods across days post-injury revealed no significant treatment effect (*p* > 0.1) on average daily MAP and HR values, but there was a significant effect of days post-injury on both MAP [*F*_(1, 21)_ = 7.282, *p* < 0.0001] and HR [*F*_(1, 21)_ = 13.23, *p* < 0.0001] values. *Post-hoc* analyses revealed a significant (*p* < 0.05) decrease in MAP values at 1 day post-injury (DPI) compared to pre-injury values, as well as compared to 3 and 4 DPI. The ensuing MAP values between 5 and 13 DPI were again significantly (*p* < 0.05) lower compared to pre-injury. Notably, however, from 14 to 22 DPI there were significant (*p* < 0.05) elevations of daily MAP compared to 5–13 DPI that approximated pre-injury values. For accompanying HR values, there were significant (*p* < 0.05) decreases from 8 to 13 DPI compared to 1 DPI which was followed by significant (*p* < 0.05), diametrically opposite increases in HR values from 14 to 22 DPI compared to 1 DPI. However, there was no significant treatment by days effect on either daily MAP [*F*_(1, 21)_ = 0.865; *p* = 0.636] or HR [*F*_(1, 21)_ = 0.383; *p* = 0.994]. Overall, there appeared to be a conspicuous elevation in both MAP and HR values beginning two weeks post-injury that remained elevated, approaching pre-injury values; the significance of this alteration is uncertain, although it does correspond to the development of AD in this model.

### Acute, not chronic GBP treatment reduces induced autonomic dysreflexia and tail muscle spasticity

As detailed in “Materials and Methods” and depicted in Table 1, on days 15 and 22, the animals receiving GBP every morning were injected with a single dose of saline (acute saline) 1 h prior to assessments, whereas the animals receiving saline every morning were given a single dose of GBP (acute GBP) 1 h prior to assessments. It was found that only acute GBP treatment significantly reduced CRD-induced MAP increases by approximately two-fold compared to acute saline treatment, irrespective of chronic morning treatments. Bradycardia also appeared reduced with GBP at all time points examined, but variability precluded significant differences. As detailed in “Materials and Methods” and depicted in Table 2, when tail spasticity was assessed in the same injured animals in response to light touch, stretch and noxious pinch, only acute GBP treatment had a significant and striking effect in virtually abolishing all three measures of tail spasticity.

### Effect of daily GBP treatment on the incidence of spontaneous autonomic dysreflexia

**Figure 3:**
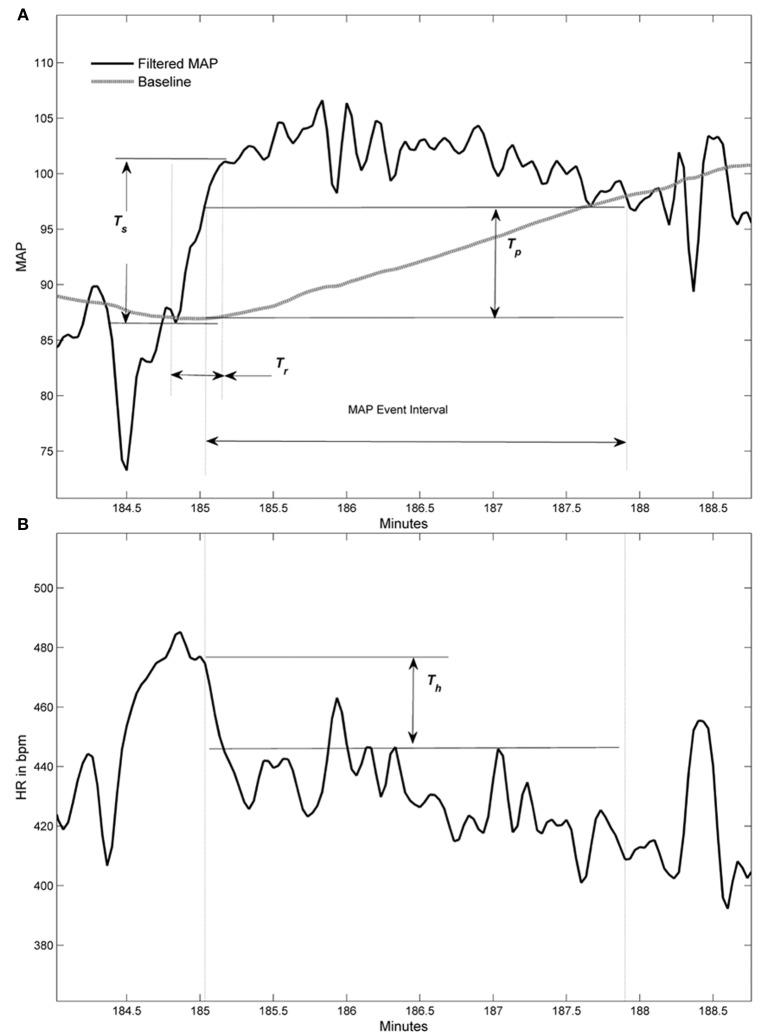
**Graphic examples of conditions used to detect an autonomic dysreflexia (AD) event. (A)** Filtered mean arterial pressure (MAP) signal with baseline (gray line) and three thresholds used in conditions for event. **(B)** Corresponding filtered heart rate (HR) signal within MAP event interval shows threshold for HR drop condition. Heart rate signal does not have a baseline.

We developed a novel algorithm to detect spontaneous events of AD based on the 24 h MAP and HR telemetry data. The algorithm was developed and thresholds determined from a sample data set with AD events labeled by human observers of the MAP, baseline MAP, and HR from several animals (about 60 h of data). An illustration of a detected AD event is shown in Figure 3. To establish the algorithm relative to human identified events, the algorithm was run on an independent set of test data where AD events were identified by human observers (see “Materials and Methods,” Statistical analyses). Results showed an agreement of greater than 80% with humanly detected events. For the times corresponding to no humanly detected AD events, the algorithm detected events at a rate of 0.0004 events per second, which corresponds to about 1 event every 42 min.

**Figure 4:**
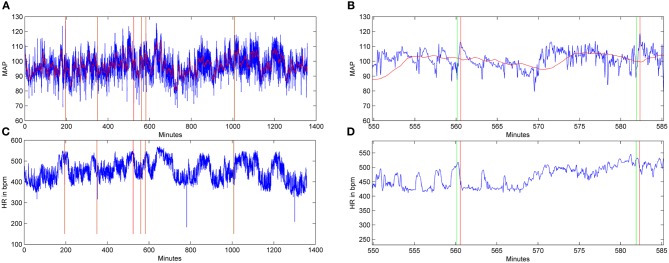
**Graphic examples of autonomic dysreflexia (AD) events detected over a 24-h period in an injured animal, employing the algorithm.** Signals for mean arterial pressure (MAP) **(A,B)** and heart rate (HR) in beats per minute **(C,D)** from a saline-treated, injured rat 15 days following T4 spinal cord transection. In the MAP and HR traces, the horizontal red lines represent the baseline trace, while the blue lines correspond to the raw signals. The HR signal does not include a baseline, only the raw blue traces. In **(B)** and **(D)**, shown at higher magnification, the green lines indicate the beginning of an AD event, while red lines coincide with the end of the event.

Through validation of humanly observed events that the program detected (indicated by green and red lines in Figure 4), the optimal parameters which corroborated humanly observed events were subsequently used to calculate spontaneous events of AD in both treatment groups over days post-injury. Notably, when the pre-injury data was analyzed with these algorithm parameters, event detections were infrequent (1–3 events/day in the 4 days prior to injury) relative to post-injury detections (5–30/day). This infers that the algorithm detects aberrant physiology rather than typical MAP and HR dynamics.

**Figure 5:**
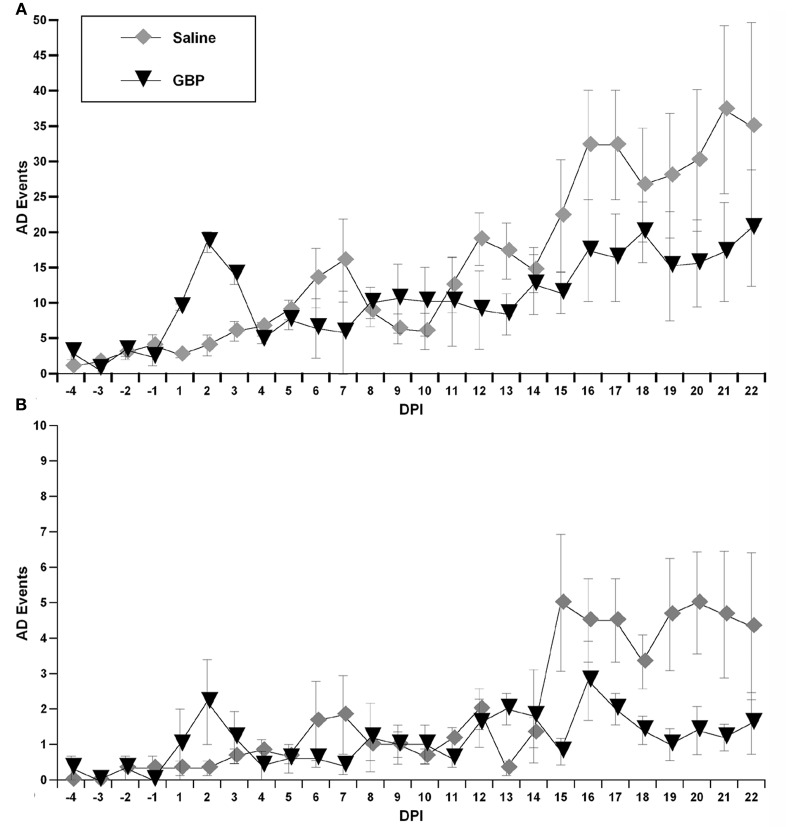
**Using the algorithm described in “Materials and Methods,” these graphs illustrate detected autonomic dysreflexia (AD) events for each chronic (daily) treatment group over 24 h (A) versus the first 4 h (B) following daily injections of gabapentin (GBP) or Saline.** Although there appeared to be fewer overall spontaneous AD events following GBP treatment at later days post-injury (**DPI**), variability precluded significant differences. Symbols represent group means ± SEM error bars.

When the algorithm was applied to the data sets from both treatment groups, it calculated the number of detected spontaneous AD events over days post-injury (Figure 5). In particular, it was used to generate both the number of detected AD events over 24 h periods between the chronic saline and GBP treatment groups (Figure 5A), as well as the number of detected AD events in the first 4 h following daily saline and GBP injections (Figure 5B). In both cases, negligible events were detected prior to injury, as expected based on the algorithm parameters. In the first several days following SCI, there appeared to be inexplicable detected AD events only in the GBP treatment group. Subsequently, however, until two weeks post-injury, the detected AD events remained only marginally elevated from pre-injury values for both groups. After 14 days post-injury, there appeared to be increases in the number of AD events detected in both treatment groups compared to pre-injury values; over 24 h or 4 h after daily injections. When a repeated measures ANOVA was run between treatment groups and the numbers of detected AD events across 22 days post-injury, there was no significant treatment effect (*p* > 0.1). When the data between treatment groups was collapsed and assessed similarly from 14 days post-injury onwards, a time when severe AD is known to be present, there was still no significant treatment effect (*p* > 0.1) over the 24 h or first 4 h periods (Figures 5A,B). While there was a significant effect of days post-injury for 24 h period values [*F*_(1, 8)_ = 2.921; *p* < 0.01], this was not the case for the first 4 h post daily injection [*F*_(1, 8)_ = 1.170; *p* = 0.329]. Accordingly, there was no significant treatment by days interaction for AD events over 24 h periods [*F*_(1, 8)_ = 0.974; *p* = 0.463] (Figure 5A) or during the first 4 h after daily injection [*F*_(1, 8)_ = 1.67; *p* = 0.121] (Figure 5B).

### Spectral analysis of blood pressure

**Figure 6:**
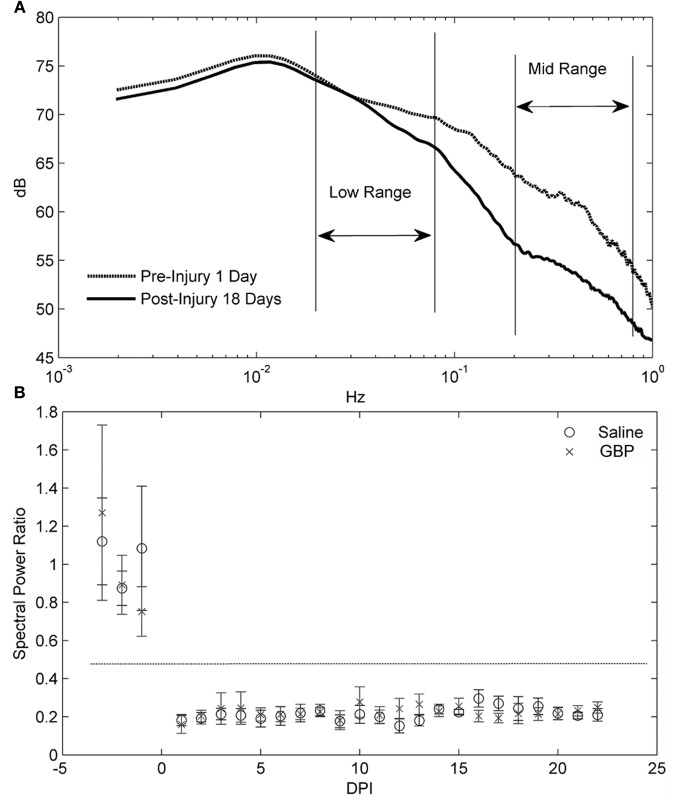
**(A)** Power spectral density (PSD) analysis averaged over all animals for one day pre- and 18 days post-injury (DPI). The key difference resulting from the injury is the loss of mid frequency (Hz) dynamics. These differences were typical of the others days as well. **(B)** For day-by-day comparisons, an average ratio between the power in low frequency range (0.02–0.08) and mid frequency range (0.2–0.8) was computed for each group. A decrease in this ratio represents a shifting of power to the lower frequencies. The results are presented with 95% confidence limits for the error bars. Note that the horizontal dashed line indicates that the 95% intervals between pre-and post-injury are non-overlapping. The relative loss in mid frequency dynamics over DPI is clearly seen by the distance between means from the pre-injury cases. However, there is no consistent difference between treatment groups over DPI.

We then sought correlations between the visible reductions of AD events in GBP-treated animals and trends in the spectral analysis of blood pressure. Critically, traditional spectral analyses do not pick up this clinically relevant event, highlighting the importance of the parameters we used to define an AD event in the algorithm. As depicted in Figure 6, spectral analysis of blood pressure only demonstrated the significant loss of a regulatory mechanism after SCI with dynamics in the 0.2–0.8 Hz (Mid) range (Figure 6A), with no consistent differences between treatment groups. Importantly, this does not negate the AD detections found using the algorithm and suggests that GBP did not impact the persistent changes resulting from the injury. The error bars in Figure 6B represent the 95% confidence limits of the mean power (PSD) ratio estimates from each group on each day. Note that for each day post-injury, the 95% confidence limits overlap; however for the pre- and post-injury days their 95% confidence limits do not overlap, indicating a significance difference (*p* < 0.05).

## Discussion

Here we report the results of long-term radio-telemetry experiments designed to determine, firstly, whether daily GBP administration for 3 weeks post-SCI versus saline altered baseline cardiovascular parameters, as well as AD elicited by noxious CRD. Notably, the same injured animals were also assessed for induced tail muscle spasticity, both distinct debilitating secondary reflexes following SCI that are often triggered by noxious stimuli below the SCI level (Rabchevsky and Kitzman, 2011). Daily GBP administration did not have significant effects on cardiovascular parameters, but there were trends for elevated MAP and HR compared to saline, notably in the first two weeks post-injury. On the contrary, and in support of our recent findings (Rabchevsky et al., 2011), acute GBP treatment significantly attenuated experimentally induced AD and tail spasticity, irrespective of chronic daily morning treatments.

GBP is currently provided as a prophylactic for neuropathic pain, indicating chronic administration. Therefore, we sought to determine whether GBP can be effectively taken orally near the onset of AD (acutely) to alleviate the debilitating reflexes. GBP possesses multiple cellular mechanisms and demonstrates the potential to help decrease the manifestation of spasticity in the chronic SCI population (Gruenthal et al., 1997; Priebe et al., 1997). Inhibition of glutamatergic transmission may be pre-eminent in mediating its therapeutic effects in epilepsy, neuropathic pain, and spasticity (Wheeler, 2002). Specifically, gabapentin has been shown to inhibit presynaptic glutamate release (Shimoyama et al., 2000; Maneuf and McKnight, 2001; Maneuf et al., 2004; Coderre et al., 2005, 2007). Moreover, glutamatergic neurotransmission has been shown to contribute to spinal viscerosympathetic initiation of episodic hypertension during experimental AD (Maiorov et al., 1997a) as well as induced tail muscle spasticity (Kitzman et al., 2007). Alternatively, GBP has been reported not to work by modulating presynaptic Ca^2+^ channel release; instead, it serves as a thrombospondin receptor which, when bound, inhibits new synapse formation in a murine model of whisker barrel de-afferentation (Eroglu et al., 2009). However, the latter study reported that GBP did not eliminate established synapses, inferring that inhibition of synapse formation with chronic daily GBP treatment cannot explain the mechanisms by which acute GBP treatments were effective in ameliorating nociceptive spinal reflexes after SCI.

Based on this and our previous findings (Rabchevsky et al., 2011), our initial expectation was that daily GBP may lower basal MAP, in part, by reducing the incidence of spontaneous AD. In order to address this intriguing possibility, we developed an algorithm to detect spontaneous events of AD based on 24 h MAP and HR data gathered over 3 weeks. Once validated, we found that chronic GBP treatment reduced daily spontaneous AD events and, more prominently, within the first 4 h after administration. The appearance of AD events and, accordingly, the suppressive effects of GBP became apparent two weeks after SCI, which corresponds to the time course of AD development (Krassioukov and Weaver, 1995; Mayorov et al., 2001; Rabchevsky, 2006). Interestingly, we observed remarkable cardiophysiological alterations in mean daily MAP and HR values in both treatment groups that began to appear following initial CRD trials on days 14–15. While, we do not have data for control injured rats, this suggests that noxious CRD may lead to profound adaptations in the spinal cord of animals with complete SCI, irrespective of treatment.

Several weeks following experimental SCI, CRD induces a rapid increase in MAP that are usually accompanied by varying degrees of bradycardia; the clinical definition of AD (Karlsson, 1999). Critically, such AD episodes can last as long as the noxious CRD is applied and the magnitude of MAP increases is typically in the range of 20–50 mm Hg above baseline, as shown in this and previous studies (Krassioukov and Weaver, 1995; Cameron et al., 2006; Hou et al., 2008). This is a key distinction between detecting spontaneous AD events versus those elicited by experimental CRD, the latter of which establishes a stable baseline MAP prior to assessments. Indeed, in developing the AD algorithm we could not find persistent prolonged MAP increases accompanied by bradycardia, most notably ≥15 mm Hg above baseline established by extended averaging windows and low-pass filtered MAP signals. It is uncertain whether the sparse transient AD events detected are a reflection of either fecal impaction or distended bladder, but the same injured rats responded to noxious CRD with significant AD, except for those receiving GBP acutely before assessments. Importantly, however, the spontaneous AD events detected over days post-injury were not significantly altered by daily, chronic GBP treatment.

Based on seminal rodent AD modeling studies (Krassioukov and Weaver, 1995; Rivas et al., 1995; Maiorov et al., 1997b; Krenz et al., 1999; Mayorov et al., 2001; Marsh et al., 2002), our own published reports have consistently reported CRD-induced changes in MAP versus changes in systolic arterial blood pressure (SAP). To our knowledge, only recently have SAP changes been reported as a primary indicator of experimentally-evoked AD (Inskip et al., 2012). Nevertheless, we reanalyzed our data sets for the current and other ongoing studies to establish the applicability of reporting changes in SAP values instead of MAP. While the magnitudes of CRD-induced SAP increases were found to be greater than MAP changes, the patterns and statistical differences were unaltered. Accordingly, we have reported the MAP changes evoked by CRD as well as spontaneous AD events detected with the algorithm.

Alternatively, it is documented in other reports, experimental and clinical, that AD episodes can also be accompanied by tachycardia, and we have observed many instances of such occurrences in this and previous studies. However, regarding the algorithm, and in line with all our previous reports, we have operationally defined AD as a MAP increase concomitant with bradycardia (Krassioukov and Weaver, 1995; Rivas et al., 1995; Karlsson, 1999). It is also appreciated that based upon our required bradycardia inclusion, we may have missed otherwise detected AD episodes. While we did observe instances of tachycardia during CRD-induced MAP increases (see Table 1), the overall HR changes ranged from −60 to +4 bpm. Accordingly, we cannot predict how many spontaneous ≥10 mm Hg increases were accompanied by tachycardia since the algorithm parameters were not set to capture such events. Despite this caveat, such a limitation is based on the user-defined parameters applied to the algorithm and, therefore, speaks to its broad applicability. For example, to re-define an AD event the algorithm can be modified to detect any chosen supra-threshold MAP increase for any given duration, but when accompanied by either tachycardia, bradycardia or both.

We chose 50 mg/kg dosage (i.p.) for this study since we already documented that this dosage and route given acutely completely eliminated spasticity and significantly abrogated CRD-induced AD in spinal rat models (Kitzman et al., 2007; Rabchevsky et al., 2011). This dosage is on the low side of what has been employed in rat models of neuropathic pain, some up to 300 mg/kg (Yoon and Yaksh, 1999; Coderre et al., 2007), and in mice it has been shown that up to 1000 mg/kg does not affect motor performance (Czuczwar et al., 2003). Also, there is no reported change in Roto-rod performance one hour after each of four consecutive daily injections of 100 mg/kg GBP (i.p.), and there are no reported differences in the half-life between GBP administered i.v. or i.p. (Xiao et al., 2007). The issue that remains unresolved, therefore, is whether a once daily injection of GBP is sufficient to have potential effects on spontaneous daily AD events detected by the algorithm.

The AD event detection algorithm has the advantage of consistently applying the same rules for every case, and while the human observers are aware of these rules, their ability to recognize the signal dynamics is limited by the need for sufficiently large swings in HR and MAP (relative to surrounding signals) to catch their attention. Therefore, the additional AD events captured by the algorithm were events that fit the definition, but were missed by the human observers either due to fatigue or the signal changes too close to the thresholds. Notably, there were insignificant detections in pre-injury data, validating injury-induced cardiovascular alterations. The missed AD events by the algorithm on humanly detected events were due, in part, to human error where a large jump in HR or MAP may have swayed a decision when both signals did not meet the criteria. On the other hand, there were cases where some transients were not entirely filtered out causing spurious threshold crossing and shortening event intervals. Humans would typically overlook these crossings, especially if other signal cues suggested the AD event was occurring. In summary, the algorithm showed good agreement with humanly identified events and provided a repeatable method for determining number of AD events, not dependent on human subjectivity or variability, and thus a useful tool when large amounts of data are available.

Although we found that daily GBP after SCI insignificantly elevated both MAP and HR values compared to daily saline treatment, notably between 1 and 2 weeks post-injury, in the current study design we did not include a sham injured GBP-treated group to determine its influences on MAP and HR compared to sham injured vehicle-treated rats. Importantly, however, we did analyze pre-injury data for each animal, which served as internal baseline controls for all cardiophysiological outcome measures in both injured treatment groups. Accordingly, such controls ensured reliability and validity of spectral analyses. The loss in the MAP fluctuations in the mid frequency range after SCI were shown to be significant, as demonstrated by the PSD comparisons of Figure 6. This strongly suggests that the regulatory mechanisms lost after SCI generate MAP fluctuations with periods on the order of 1.25–5 s (0.2–0.8 Hz). It is worth noting that comparison of spectra before and 18 days post-injury (Figure 6A) showed differences in the Mid frequency range, a region that has been associated in rat with sympathetically mediated effects upon blood pressure variability (Brown et al., 1994; Julien et al., 2003). Importantly, similar blood pressure fluctuations must occur within many intervals used in PSD estimation to detect such events. In other words, the blood pressure signals from the AD events were not sufficiently stationary to emerge in the spectral analysis and, accordingly, such analyses were blind to sparse transient events that we defined as AD. This stresses the importance and significance of detecting transient irregular AD events independently of spectral analysis, since frequency domain analyses are restricted to identify activity that is persistent over the observation epoch. Such analyses do indicate, therefore, that GBP does not affect the persistent dynamics of MAP related to SCI.

In summary, we designed a preclinical study to develop a treatment for chronic SCI individuals who continually suffer from secondary complications, notably abnormal muscle spasms and autonomic spinal reflexes. There is currently no pharmaceutical intervention which is known to effectively attenuate neuropathic pain as well as the manifestation of both muscular spasticity and AD after chronic SCI. While the clinical significance of the persistent MAP fluctuations associated with SCI is unclear, transient AD events directly impact the individual with SCI and, therefore, analyses directed at detecting and characterizing these event are critical for assessing treatments for SCI patients. Consequently, this quantitative experimental study was requisite to establish proof-of-principle prior to direct clinical application employing a two-pronged approach designed to alleviate dissimilar aberrant neurologic reflexes with a single drug, GBP. Such preclinical data appears to support the novel indication of GBP for the treatment and maintenance of both spasticity and AD following SCI.

### Conflict of interest statement

The authors declare that the research was conducted in the absence of any commercial or financial relationships that could be construed as a potential conflict of interest.
